# Effect of alpha-mangostin on olanzapine-induced metabolic disorders in rats 

**DOI:** 10.22038/IJBMS.2022.58734.13047

**Published:** 2022-02

**Authors:** Alireza Ardakanian, Mahboobeh Ghasemzadeh Rahbardar, Farzaneh Omidkhoda, Bibi Marjan Razavi, Hossein Hosseinzadeh

**Affiliations:** 1 Department of Pharmacodynamics and Toxicology, School of Pharmacy, Mashhad University of Medical Sciences, Mashhad, Iran; 2 Targeted Drug Delivery Research Center, Pharmaceutical Technology Institute, Mashhad University of Medical Sciences, Mashhad, Iran; 3 Pharmaceutical Research Center, Pharmaceutical Technology Institute, Mashhad University of Medical Sciences, Mashhad, Iran

**Keywords:** Anti-oxidants, Leptin, Liver, Mangostin, Metabolic syndrome, Obesity, Olanzapine, Weight gain

## Abstract

**Objective(s):**

As olanzapine has side effects such as weight gain and metabolic disorders, and alpha-mangostin has been shown to control metabolic disorders, the effects of alpha-mangostin on metabolic disorders induced by olanzapine were investigated in this study.

**Materials and Methods:**

Obesity was induced in female Wistar rats by daily administration of olanzapine (5 mg/kg/day, IP, 14 days). Rats were divided into 6 groups:1) vehicle (control); 2) olanzapine (5 mg/kg/day); 3,4,5) olanzapine+ alpha-mangostin (10, 20, 40 mg/kg/day, IP); 6) alpha-mangostin (40 mg/kg/day). Weight changes were measured every 3 days and food intake was assessed every day. Systolic blood pressure, plasma levels of blood sugar, triglycerides, total cholesterol, HDL, LDL, leptin, oxidative stress markers (MDA, GSH), AMPK, and P-AMPK protein levels in liver tissue were assessed on the last day of the study.

**Results:**

Administration of olanzapine significantly increased weight gain, food intake, blood pressure, triglycerides, LDL, blood sugar, leptin, and MDA in rat liver tissue and also decreased GSH, AMPK, and P-AMPK in liver tissue compared with the control group. Different doses of alpha-mangostin significantly reduced weight gain, food intake, systolic blood pressure, triglycerides, LDL, blood sugar, leptin, and MDA. Also, they significantly increased GSH, AMPK, and P-AMPK in liver tissue compared with the olanzapine group.

**Conclusion:**

Olanzapine increases leptin levels, food intake, and weight, induces oxidative stress, decreases the levels of AMPK and P-AMPK proteins in liver tissue, and causes metabolic disorders. But, alpha-mangostin reduces the negative effects of olanzapine by activation of AMPK.

## Introduction

Metabolic syndrome, as a metabolic disorder, includes a variety of symptoms such as increased food and glucose intake, atypical serum levels of fats, raised triglycerides (TG), decreased high-density lipoprotein cholesterol (HDL-C) levels, abdominal obesity, overweightness, insulin resistance, and high blood pressure which might result in type 2 diabetes, cancer, cardiovascular disease, short life, besides poor quality of life (1-3). The pathophysiology of the complications of metabolic syndrome is probably due to an imbalance in calorie and energy intake, along with changes in genetics and lifestyle (4-6). Metabolic syndrome is a threat to human health that requires immediate prevention and treatment, thus effective strategies must be implemented to reduce the burden of this disease (7-9).

Olanzapine, a derivative of thienobenzodiazepine, was patented in 1971 and approved in 1996 in the United States for medical use, including to reduce the symptoms of schizophrenia, bipolar disorder, and cognitive problems. Olanzapine belongs to a quite new group of second-generation antipsychotics, also known as atypical antipsychotics. Though there is no complete characterization of atypical antipsychotic medicines, in general, unlike typical antipsychotics, they are more prone to 5-HT_2A _serotonin receptors than dopamine D_2_ receptors, have fewer extra-pyramidal symptoms, and amend negative indications (10, 11). 

The most important side effects of olanzapine are increased appetite (12), hyperlipidemia (13, 14), significant weight gain (15), insulin resistance (16), decreased insulin signaling in muscles and liver (17), impaired glucose homeostasis (18), and increased blood glucose (19), which ultimately raises the possibility of type 2 diabetes (20), as well as heart-metabolic disorders (17). The prevalence of obesity in patients who have been using this drug for more than 4 weeks has been reported to be 50% (21, 22). Also, obesity exacerbates the symptoms of mental illness and reduces life expectancy in patients by 15–20 years (23). Some previous studies have also shown that administration of olanzapine (5 mg/kg, 15 days) to rats increased systolic blood pressure (24). In another study, female rats that received olanzapine (1–2 mg/kg) twice daily for 20 days showed a significant increase in systolic blood pressure (25). 

AMP-activated protein kinase (AMPK), mediated by the H_1_ receptor, is the main mechanism in the progress of antipsychotic-induced hyperphagia (26). AMPK, a cell-level energy homeostasis sensor, integrates metabolic signals and regulates energy balance by modulating fatty acid metabolism in the hypothalamus (27).

Nowadays, plants are an important source of medicines. Many of the medicinal plants and their active ingredients have been widely used by scientists to protect cells from damage.

Mangosteen (*Garcinia mangostana* L.) is a juicy reddish-purple fruit with a pleasant taste that is common in Southeast Asian countries such as India, Myanmar, Thailand, Sri Lanka, Malaysia, and the Philippines, and its pericarp is used in folk medicine to manage chronic intestinal disorders, respiratory disorders, trauma, diarrhea, skin infections, also ulcers (28-30). In 1855, alpha-mangostin was identified as one of the major xanthones of mangosteen pericarp (31). This compound is a yellow substance that can be obtained from other parts of the plant, such as dried sap and tree bark (32). Alpha-mangostin has many medicinal properties, including antibacterial, antifungal, anti-parasitic, anti-obesity, anti-diabetic, anti-cancer, antioxidant, and anti-inflammatory properties (33-40).

It has been observed that the administration of alpha-mangostin (50 mg/kg, p.o., 6 weeks) to obese rats on a high-fat diet significantly reduced body weight, fat mass, serum cholesterol, TG, and fatty acids. Also, it has been suggested that alpha-mangostin exhibits its anti-obesity effects by activating hepatic expression of AMPK, sirtuin 1 (SirT1), and peroxisome proliferator-activated receptor γ (PPARγ) (41). Furthermore, several studies in diabetic rats have shown that administration of alpha-mangostin (100–200 mg/kg) by gavage for 8 weeks reduced blood pressure and mean arterial pressure (42, 43).

Since many patients suffering from bipolar disorder and schizophrenia receiving olanzapine are vulnerable to weight gain and its adverse consequences, and alpha-mangostin is known to be an advantageous factor in controlling and amending these metabolic disorders, in this study the effect of alpha-mangostin (10, 20, 40 mg/kg, IP, 14 days) on metabolic disorders caused by olanzapine (5 mg/kg, IP, 14 days) in rats was investigated by evaluating weight changes (every 3 days) and food intake (every day). Besides, systolic blood pressure, plasma levels of blood sugar, TG, total cholesterol (TC), high-density lipoprotein (HDL), low-density lipoprotein (LDL), leptin, oxidative stress markers (malondialdehyde (MDA), glutathione (GSH)), AMPK, and P-AMPK proteins in liver tissue were assessed on the last day of the study. 

## Materials and Methods

***Animals***

Female Wistar rats weighing an average of 180 g were used in this study. The rats were procured from the animal breeding room of Mashhad School of Pharmacy and were kept in cages in the temperature range of 22–25 °C, 12/12 hr light /dark cycle, without the restriction of access to water, and 50 g of daily food. However, to measure factors such as blood sugar and blood lipids they had to have fasted, thus, the animals’ access to food was cut off on the last night of the study for 12 hr before taking the blood sample. Also, all animal experiments were performed as stated by the procedures of the ethics committee of Mashhad School of Pharmacy (code 958932). These rules are also the same as the Internationally Accepted Principles for Animal Use and Care (44).

***Materials***

Olanzapine was purchased from Sobhan, Iran. Alpha-mangostin was attained from Trademax Pharmaceuticals & Chemicals Co, China (purity >90%). The other chemicals were acquired as follows: acetic acid (Merck, Germany, cat#10056), Bovine serum albumin (BSA) (GoldBio, USA, cat#A-420-10), protease inhibitor cocktail (Sigma, Germany, P8340), dry skim milk (Quetlab, UK, cat# QB-65-5250), 2-mercaptoethanol (Merck, Germany, cat#15433), ethylene glycol tetraacetic acid (EGTA) (Sigma, USA, cat#E3889), NaF (Merck, Germany, cat# B320341), ethylenediaminetetraacetic acid (EDTA) (Pars Tous Biotechnology, Iran, cat# B111061), sodium deoxycholate (Sigma, New Zealand, cat# D6750), Tris (Merck, Germany, cat#1083870500), sodium orthovanadate (Na3VO4) (Sigma, Madhya Pradesh, India, cat#S6508), Sodium Dodecyl Sulfate (SDS) (Merck, Germany, cat#817034), sodium-β glycerophosphate (Sigma, Germany, cat#50020), phenylmethylsulfonyl fluoride (PMSF) (Sigma, Germany, cat#78830), Triton X-100 (Merck, Germany, Art. 11869 ), ethanol (Merck, Germany, cat#107017), and polyvinylidene difluoride (PVDF) (Bio-Rad, USA, cat#162-0177).

***Study design***

The experiments were performed on 36 female Wistar rats, which were divided into 6 groups (n=6). For 14 days, all injections were given intraperitoneally once a day.

1: Animals receiving alpha-mangostin solvent plus olanzapine vehicle

2: Animals receiving olanzapine (5 mg/kg/day) (24);

3: Animals receiving olanzapine (5 mg/kg/day) with alpha-mangostin (10 mg/kg/day);

4: Animals receiving olanzapine (5 mg/kg/day) with alpha-mangostin (20 mg/kg/day) (45);

5: Animals receiving olanzapine (5 mg/kg/day) with alpha-mangostin (40 mg/kg/day) (45);

6: Animals receiving alpha-mangostin (40 mg/kg/day).

Olanzapine was dissolved in normal saline (NS) 0.9% and 2 drops of acetic acid, and alpha-mangostin was dissolved in NS and tween 80 (70 μl in each cc). 

Selected doses of alpha-mangostin were in an innocuous range as previous research has shown that oral administration of alpha-mangosteen at 100 mg/kg per day for 30 days did not cause any toxicity in mice, did not alter blood factor levels, and it was tolerable (35). In another study, the mean intraperitoneal lethal concentration (LC_50_) of alpha-mangosteen in mice was obtained at (150 mg/kg) (36). Alpha-mangostin toxicity was also studied in young rats and it was reported that there were no significant adverse effects up to dose of 80 mg/kg (46).

***Measuring food intake and weight changes***

Throughout the study, food intake was tracked regularly (every day), and body weight was measured every three days. The daily food consumption was calculated by calculating the difference between the total amount of food in the box and the leftovers after 24 hr. The food efficiency ratio (FER) was measured by the equation: FER= body weight gain (g/day)/food intake (g/day) 

***Measuring systolic blood pressure***

The rat tail’s systolic blood pressure was measured using a non-invasive tail-cuff method (25). An LED light was used to clean and warm the rat’s tail. By pressing the corresponding key on the blood pressure monitor (NIBP Controller), air enters the blood pressure cuff and the heart rate returns to normal after a short time (a few seconds). The systolic blood pressure is the point at which the animal’s heartbeat resumes and the pulse amplitude returns to normal. The mean blood pressure was used to calculate the final systolic blood pressure after measuring the systolic blood pressure five times.

***Measuring the amounts of serum glucose, TG, TC, HDL, and LDL***

After the injection period, the overnight fasted rats (10–12 hr) were sacrificed. Then the serum samples were sent to the laboratory to measure the amounts of glucose (Pars Azmoon, Iran, cat# 98007), TG (Pars Azmoon, Iran, cat# 98003), TC (Pars Azmoon, Iran, cat# 98004), HDL (Man Co, Iran, cat#180911), and LDL amounts using enzymatic kits with a clinical spectrophotometer (Hitachi, Japan).

***Measuring the leptin amount in serum***

The amount of leptin in blood serum samples was calculated using an enzyme-linked immunosorbent assay (ELISA) kit (Shanghai Crystal Day Biotech) based on biotin double antibody sandwich technology and the kit protocol. This kit is based on the specific relationship between antibodies and leptin. The blank solution, various standard dilutions, and samples of different groups were poured into the wells. The leptin in the samples binds to the antibody attached to the plate wall and was incubated at 37 °C for 60 min. The wells were then washed five times with the washing solution, each time for 30 sec. In the next step, chromogen solutions were added to the wells and incubated again for 10 min. After this step, a yellow color was visible which turned to blue by adding the final solution. Finally, the color intensity was read with an ELISA reader at 450 nm and the amount of leptin in the samples was calculated and reported with a standard graph.

***Measuring the amount of malondialdehyde (MDA) in liver tissue***

This method is based on color spectrophotometric measurements generated from the progression of the thiobarbituric acid (TBA) reaction with MDA.

At the beginning of the experiment, 200 mg of each liver sample is placed in a 5 ml micro-tube (placed in ice) and by adding 1.15% KCL, 10% tissue homogenate is made. Then, tissue homogenate is vortexed. 500 µl of homogenate tissue is poured into a glass tube and 3 ml of 1% phosphoric acid and 1 ml of 0.6% TBA are added and mixed. After that, glass tubes are placed in boiling water for 45 min. Then 4 ml of butanol is added to each cooled sample and vortexed for 1 minute. Next, the samples are centrifuged for 15–20 min so that the top liquid is completely pink. The absorption rate of the organic phase of the samples is read at 532 nm (blank is butanol). The standard curve is plotted in the concentration range of 0–100 MDA nmol/ml to measure the MDA concentration, and then the concentrations are expressed as nmol/g tissue (47).

***Measuring the amount of glutathione (GSH) in liver tissue***

The reaction of sulfhydryl groups with the 5,5’-dithiobis-(2-nitrobenzoic acid) (DTNB) reagent, which reacts with sulfhydryl groups to form a color complex with maximum absorption of 412 nm, is the basis of this experiment.

First, 200 mg of each liver sample is placed in a 5 ml micro-tube (placed in ice), and 2 ml of phosphate buffer (pH = 7.4) is added to the sample to make 10% tissue homogenate. In the next step, 500 µl of tissue homogenate and 500 µl of 10% trichloroacetic acid (TCA) is poured into a 2 ml microtube and vortexed (all above steps are performed on ice). The samples are then centrifuged at 10,000 rpm for 6 min. Then, 500 µl of the clear supernatant is mixed with 2.5 ml of phosphate buffer (pH = 8). Then, 500 µl of DTNB reagent is added to the tubes and yellow color appears. In the end, the absorption is read at 412 nm. A standard curve is used to measure the GSH concentration, and the GSH amount is expressed as nmol/g tissue (48).

The blank solution in this experiment contains 250 µl of phosphate buffer (pH = 7.4), 250 µl of TCA, 2.5 ml of phosphate buffer (pH = 8), and 500 µl of DTNB reagent.

***Western blotting***

The liver samples were defrosted and placed in a lysis buffer containing 50 mM Tris–HCl (pH: 7.4), 2 mM EDTA, 2 mM EGTA, 10 mM NaF, 1 mM sodium orthovanadate (Na_3_VO_42_H_2_O), 10 mM glycerophosphate, 0.2% W/V sodium deoxycholate, 1 mM phenylmethylsulfonyl fluoride, and complete protease inhibitor cocktail (Roche, Mannheim, Germany). The homogenates were then sonicated on ice for three 10 Sec bursts at high strength, followed by a 10 Sec cooling cycle, and centrifuged (10,000g) for ten min at 4 °C. The Bradford assay kit (Bio-Rad; Bradford, 1976) was used to determine protein concentration and regulate sample contents. Each modified sample was mixed 1:1 v:v with 2× SDS blue buffer, boiled, aliquoted, and stored in a −80 °C freezer. Samples were loaded (50 μg of protein/lane), electrophoresed on a 12% SDS-polyacrylamide gel electrophoresis system (SDS = PAGE), and blotted to a polyvinylidene fluoride membrane (PVDF; Bio-Rad). The PVDF papers were then blocked for 2 hr in skim milk. Rabbit polyclonal anti-AMPK (Cell Signaling #3632s, 1:1,000), rabbit polyclonal anti-P-AMPK (Cell Signaling #3631s, 1:1,000), and mouse polyclonal anti-β-actin antibodies (Cell Signaling #4967, 1:1,000) were used to incubate the blots for 2 hr on a rocker. Membranes were incubated with rabbit or mouse horseradish peroxidase-conjugate anti-IgG (Cell Signaling #7071, 1:2,000; Cell Signaling #7072, 1:2,000, respectively) for 2 hr after being washed three times with Tris-Buffered Saline and Tween 20 (TBST). The peroxidase-coated bands were visualized using enhanced chemiluminescence. Alliance 4.7 Gel doc (UK) was used to calculate the integrated optical densities of bands. UV Tec software (UK) was used to perform densitometric analysis for protein bands. The protein levels were compared with the corresponding bands of the control protein β-actin.

***Statistics***

The Prism 6 software program was used to perform the statistical calculations. The findings are presented as Mean ± standard deviation (SD). To compare the different groups, one-way and two-way ANOVA, as well as Tukey–Kramer and Bonferroni post-tests were used. For the average body weight gain and average food intake, two-way ANOVA was used. A significant difference was described as a *P*-value of less than 0.05.

## Results

***The effect of olanzapine and alpha-mangostin on rat weight changes and mean food intake***

**Figure 1:**
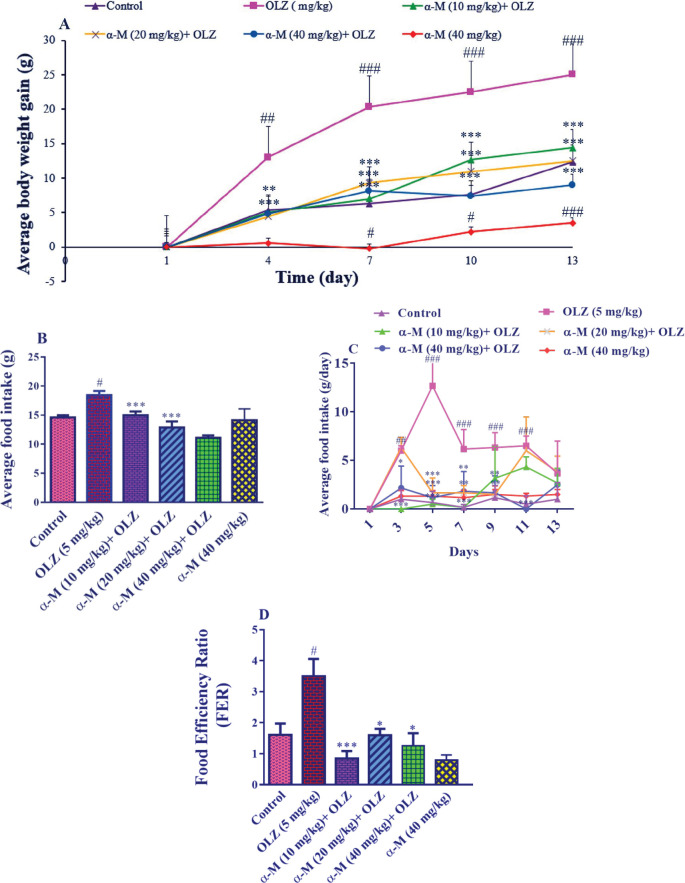
Effect of olanzapine and alpha-mangostin on A: body weight gain, B: mean total food intake, C: average food intake, and D: food efficiency ratio in rats. Olanzapine (5 mg/kg) and alpha-mangostin in different doses (10, 20, 40 mg/kg) were injected intraperitoneally into the animals. The data is reported as Mean± SD (n=6). Two-way ANOVA followed by Bonferroni* post-hoc* test for multiple comparisons (A, C) and one-way ANOVA followed by Tukey-Kramer *post-hoc* test for multiple comparisons (B, D) were used to measure the statistical difference

The results of average body weight gain in rats in different groups showed that administration of olanzapine (5 mg/kg) increased the mean weight changes in comparison with the control group, from day 4 (*P*<0.01) until days 7–13 (*P*<0.001). Also, administration of alpha-mangosteen (10 mg/kg) along with olanzapine caused weight loss compared with the olanzapine group on day 4 (*P*<0.01) and days 7–13 (*P*<0.001). Alpha-mangosteen at doses of 20 and 40 mg/kg along with olanzapine injection for 14 days was able to reduce weight in rats (*P*<0.001). Also, it was observed that injection of alpha-mangosteen (40 mg/kg) on ​​days 7–10 (*P*<0.05) and 13 (*P*<0.001) caused weight loss compared with the control group (Figure 1A).

The mean total food intake of rats in various groups showed that olanzapine (5 mg/kg) administration increased mean total food intake relative to the control group (*P*<0.05). When co-administered with olanzapine injection, alpha-mangostin (10, 20, 40 mg/kg) was able to minimize mean total food intake, which was not significant at a dose of 10 mg/kg but was significant at doses of 20 and 40 mg/kg compared with the olanzapine group (*P*<0.001). Furthermore, alpha-mangostin (40 mg/kg) did not affect the mean total food intake when compared with the control group (Figure 1B).

Furthermore, on days 3 (*P*<0.01), 5, 7 (*P*<0.001), 9, and 11 (*P*<0.001), olanzapine (5 mg/kg) increased the average food intake relative to the control group. In comparison with the olanzapine group, alpha-mangostin (10 mg/kg) administration with olanzapine decreased the average food intake on days 3, 5, and 7 (*P*<0.001). On days 5 (*P*<0.001), 7, and 9 (*P*<0.01), receiving alpha-mangostin (20 mg/kg) concurrently with olanzapine decreased average food intake in comparison with the olanzapine group. On days 3 (*P*<0.05), 5 (*P*<0.001), 7, 9 (*P*<0.01), and 11 (*P*<0.001) alpha-mangostin (40 mg/kg) in combination with olanzapine decreased the average food intake triggered by olanzapine. The sole administration of alpha-mangostin (40 mg/kg) did not affect average food intake in comparison with the control group (Figure 1C).

Furthermore, as compared with the control group, olanzapine increased FER (*P*<0.05). The FER induced by olanzapine was reduced by alpha-mangostin (10 mg/kg) (*P*<0.001) and (20, 40 mg/kg) (*P*<0.05). In contrast to the control group, alpha-mangostin (40 mg/kg) administration reduced FER (Figure 1D).

***Effect of olanzapine and alpha-mangostin on serum lipid profile and fasting blood sugar (FBS)***

The results showed that in the olanzapine (5 mg/kg) group, the level of TG was significantly higher than in the control group (*P*<0.01). Besides, receiving alpha-mangostin (10, 20, 40 mg/kg) with olanzapine injection significantly decreased serum TG levels (*P*<0.001). Also, alpha-mangostin (40 mg/kg) did not affect serum TG levels as compared with the control group.

Administration of olanzapine and alpha-mangostin had no major effect on serum HDL and TC levels in the different groups, as shown in Table 1.

In contrast to the control group, olanzapine (5 mg/kg) induced a substantial increase in serum LDL (*P*<0.05). Moreover, alpha-mangostin (10, 20 mg/kg) (*P*<0.001) and alpha-mangostin (40 mg/kg) (*P*<0.01) along with olanzapine injection reduced serum LDL levels significantly. Furthermore, as compared with the control group, injection of alpha-mangostin (40 mg/kg) did not affect serum LDL levels. Also, as compared with the control group, olanzapine (5 mg/kg) induced a significant increase in FBS (*P*<0.05). In comparison with the olanzapine group, alpha-mangostin (10, 20 mg/kg) with olanzapine was ineffective in reducing FBS. However, administration of alpha-mangostin (40 mg/kg) with olanzapine injection (*P*<0.01) reduced the amount of FBS in the blood significantly. When compared with the control group, alpha-mangostin (40 mg/kg) did not affect blood FBS levels (Table 1).

***Effect of olanzapine and alpha-mangostin on systolic blood pressure***

**Figure 2:**
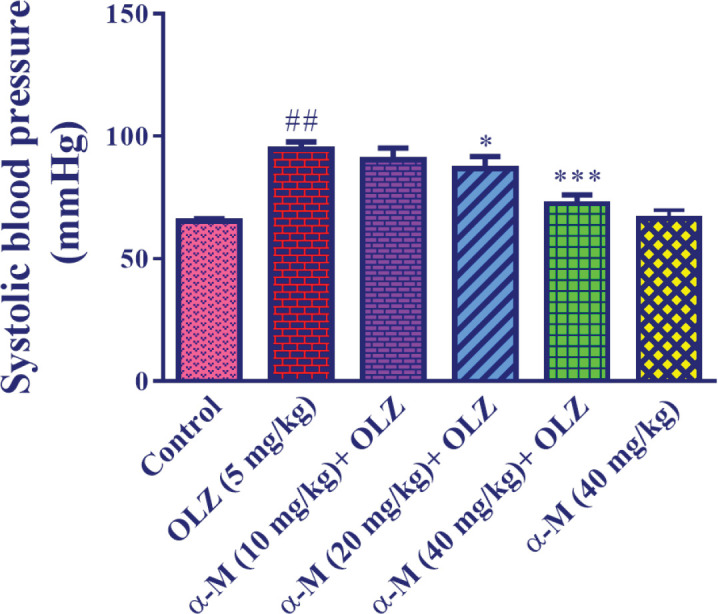
Effect of olanzapine and alpha-mangostin on systolic blood pressure. Olanzapine (5 mg/kg) and alpha-mangostin in different doses (10, 20, 40 mg/kg) were injected intraperitoneally into the animals. The data is reported in Mean± SD (n=6). One-way ANOVA test and Tukey-Kramer posttest were used to measure the statistical difference

In comparison with the control group, olanzapine (5 mg/kg) induced a significant rise in systolic blood pressure (*P*<0.01). In contrast to the olanzapine group, alpha-mangostin (10 mg/kg) with olanzapine was ineffective in lowering systolic blood pressure. However, when paired with olanzapine injection, alpha-mangostin (20 mg/kg) (*P*<0.05) and (40 mg/kg) (*P*<0.001) significantly decreased systolic blood pressure in comparison with the olanzapine group. Besides, as compared with the control group, alpha-mangostin (40 mg/kg) did not affect systolic blood pressure (Figure 2).

***Effect of olanzapine and alpha-mangostin on serum leptin levels***

For the leptin test, doses of 20 and 40 mg/kg alpha-mangostin were chosen based on previous studies.

**Figure 3:**
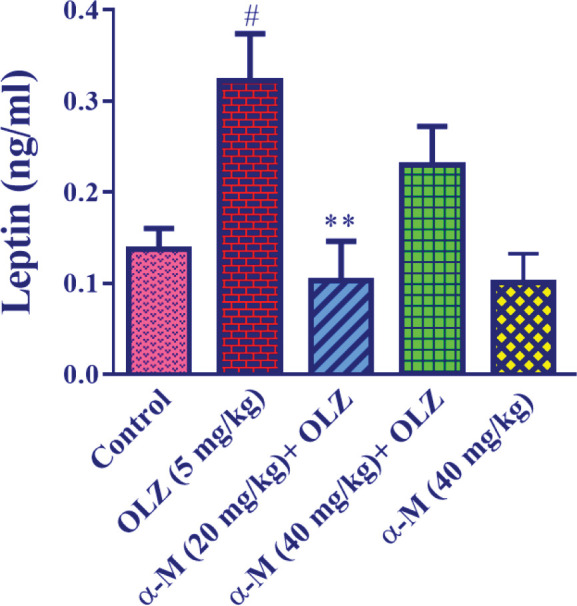
Effect of olanzapine and alpha-mangostin on serum leptin levels in rats. Olanzapine (5 mg/kg) and alpha-mangostin in different doses (20, 40 mg/kg) were injected intraperitoneally to the animals. The data is reported in Mean± SD (n=4). One-way ANOVA test and Tukey-Kramer posttest were used to measure the statistical difference

Figure 3 indicates that olanzapine (5 mg/kg) administration

resulted in a significant increase in serum leptin levels as compared with the control group (*P*<0.05). Also, when alpha-mangostin (20 mg/kg) was administered with olanzapine, serum leptin levels were significantly lower (*P*<0.01) than in the olanzapine group. In contrast to the olanzapine group, alpha-mangostin (40 mg/kg) plus olanzapine injection did not cause a substantial reduction in blood leptin. Moreover, as compared with the control group, injection of alpha-mangostin (40 mg/kg) did not affect serum leptin levels.

***Effect of olanzapine and alpha-mangostin on MDA and GSH levels of liver tissue***

**Figure 4:**
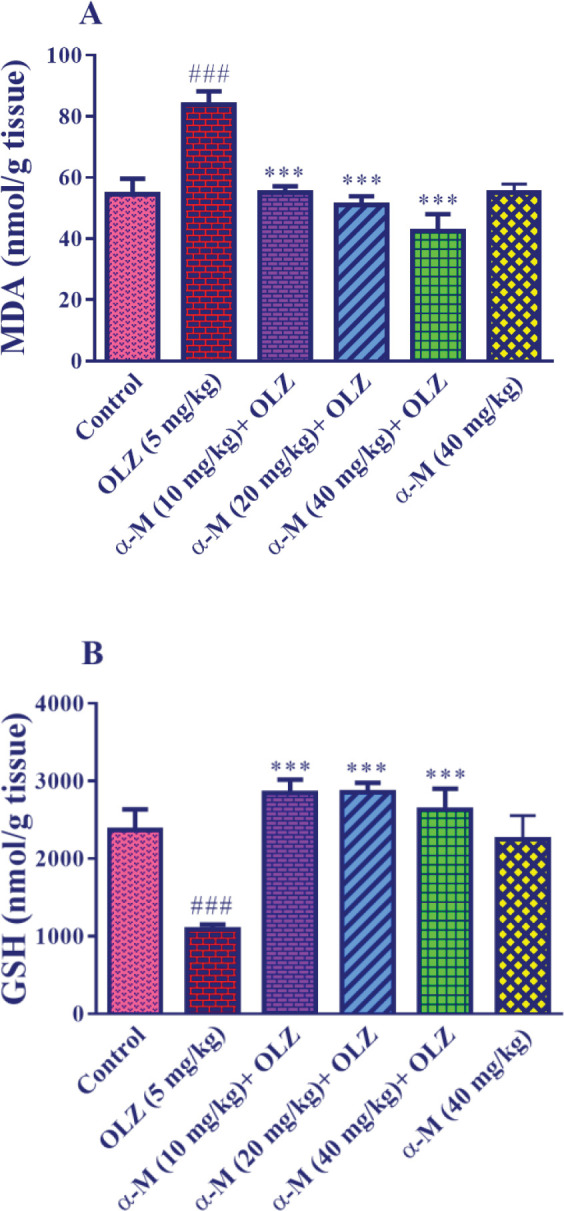
Effect of olanzapine and alpha-mangostin on liver tissue A: MDA and B: GSH levels in rats. Olanzapine (5 mg/kg) and alpha-mangostin in different doses (10, 20, 40 mg/kg) were injected intraperitoneally into the animals. The data is reported in Mean± SD (n=6). ANOVA test and Tukey-Kramer posttest were used to measure the statistical difference

Figure 4A shows that olanzapine (5 mg/kg) administration resulted in a significant increase in MDA level in liver tissue in comparison with the control group (*P*<0.001). Also, when alpha-mangostin in varying doses (10, 20, 40 mg/kg) was combined with olanzapine injection, MDA levels were significantly lower (*P*<0.001) than in the olanzapine group. Furthermore, as compared with the control group, injection of alpha-mangostin (40 mg/kg) did not affect the amount of MDA in the liver tissue.

Figure 4B indicates that olanzapine (5 mg/kg) decreased the amount of GSH in liver tissue significantly (*P*<0.001) as compared with the control group. Also, when alpha-mangostin in various doses (10, 20, 40 mg/kg) was administered along with olanzapine injection, the amount of GSH increased significantly (*P*<0.001) compared with the olanzapine group. Besides, as compared with the control group, injection of alpha-mangostin (40 mg/kg) did not affect the GSH level of liver tissue.

***Effect of olanzapine and alpha-mangostin on AMPK and P-AMPK levels in liver tissue***

For western blotting, doses of 20 and 40 mg/kg of alpha-mangostin were chosen based on previous experiments.

**Figure 5:**
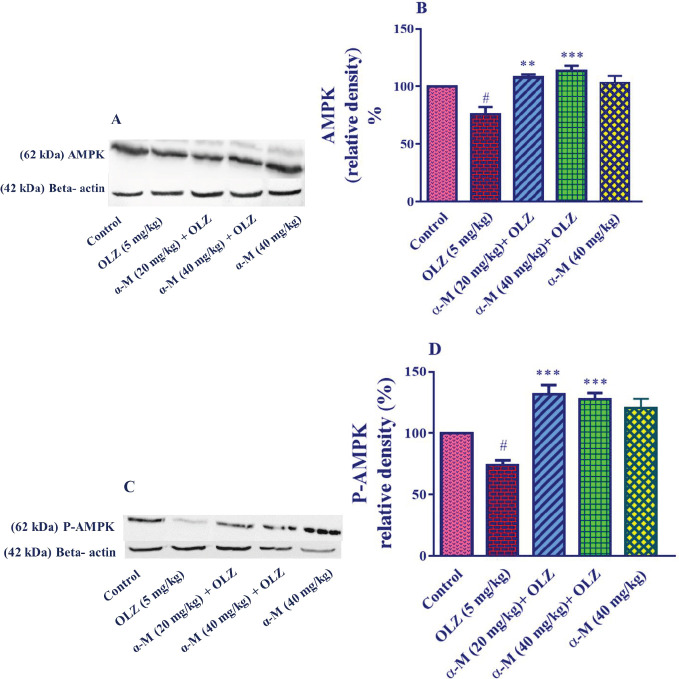
Effect of olanzapine and alpha-mangostin on liver tissue AMPK and P-AMPK levels in rats. Olanzapine (5 mg/kg), alpha-mangostin in different doses (20, 40 mg/kg) were injected intraperitoneally into the animals. The data is reported in Mean± SD (n=4). A, C: Represent immunoblot bands of the western blotting analysis. B, D: Represent quantitative presentation of the immunoblots. Data were analyzed by one-way ANOVA followed by Tukey's for multiple comparisons. Beta-actin is the loading protein control

In comparison with the control group, olanzapine (5 mg/kg) significantly decreased the amount of AMPK in liver tissue (*P*<0.05). In addition, combining administration of alpha-mangostin (20 mg/kg) (*P*<0.01) and alpha-mangostin (40 mg/kg) (*P*<0.001) with olanzapine injection resulted in a significant increase in alpha-mangostin (40 mg/kg) (*P*<0.001). However, as compared with the control group, alpha-mangostin (40 mg/kg) did not affect AMPK levels in liver tissue (Figures 5A, B).

When compared with the control group, olanzapine (5 mg/kg) significantly decreased the amount of P-AMPK in liver tissue (*P*<0.05). Also, compared with the olanzapine samples, alpha-mangostin (20, 40 mg/kg) plus olanzapine injection increased the P-AMPK amount in liver tissue (*P*<0.001). However, as compared with the control group, alpha-mangostin (40 mg/kg) did not affect P-AMPK levels in liver tissue (Figures 5C, D).

OLZ: Olanzapine, α-M: alpha-mangostin

### *P*<0.001, ## *P*<0.01, and # *P*<0.05 compared with the control group, and *** *P*<0.001, ** *P*<0.01, and * *P*<0.05 compared with the olanzapine group

**Table 1:** Effect of olanzapine and alpha-mangostin on serum lipid profile and fasting blood sugar (FBS)

	**Control**	**OLZ (5 mg/kg)**	**a-M (10 mg/kg)+ OLZ**	**a-M (20 mg/kg)+ OLZ**	**a-M (40 mg/kg)+ OLZ**	**a-M (40 mg/kg)**
**TC (mg/dl)**	81.00±2.90	91.50±5.79	64.00±2.81	65.66±2.99	63.00±4.50	64.80±2.31
**TG (mg/dl)**	119.66±8.08	418.25±78.12 ##	133.66±13.41 ***	123.33±14.47 ***	81.20±6.87 ***	103.00±18.95
**HDL (mg/dl)**	27.33±1.06	26.66±2.12	23.16±1.21	23.50±0.92	21.16±1.41	21.50±1.37
**LDL (mg/dl)**	10.66±1.01	15.50±1.33 #	7.00±0.34 ***	8.66±0.88 ***	9.40±0.72 **	8.83±0.44
**FBS (mg/dl)**	116.66±2.57	157.00±13.62 #	152.20±2.83	125.40±3.71	100.80±2.67 **	131.33±5.81

OLZ: Olanzapine, α-M: Alpha-mangostin, TC: Total cholesterol, TG: Triglyceride, HDL: High-density lipoprotein, LDL: Low-density lipoprotein, FBS: Fasting blood sugar

## *P*<0.01, # *P*<0.05 compared with the control group, and *** *P*<0.001, ** *P*<0.01 compared with the olanzapine group

OLZ: Olanzapine, α-M: alpha-mangostin

## *P*<0.01 compared with the control group, and *** *P*<0.001 and * *P*<0.05 compared with the olanzapine group

OLZ: Olanzapine, α-M: alpha-mangostin

#* P*<0.05 compared with the control group and ** * P*<0.01 compared with the olanzapine group

OLZ: Olanzapine, α-M: alpha-mangostin, MDA: malondialdehyde

### *P*<0.001 compared with the control group and *** *P*<0.001 compared with the olanzapine group

OLZ: Olanzapine, α-M: alpha-mangostin

# *P*<0.05 compared with the control group, and *** *P*<0.001 and ** *P*<0.01 compared with the olanzapine group

## Discussion

According to previous studies, atypical antipsychotic drugs, including olanzapine, cause metabolic disorders such as hyperglycemia, hypertension, weight gain, hyperlipidemia, diabetes, and cardiovascular disorders (12, 13). On the other hand, alpha-mangostin, the main xanthone of mangosteen fruit, has antioxidant, anti-obesity, anti-hypertensive, and liver-protective properties, and its effects have been considered in the treatment of metabolic syndrome (34, 49). Therefore, in the present study, the effect of alpha-mangostin on weight gain and metabolic disorders induced by olanzapine was investigated.

The obtained results revealed that receiving olanzapine (5 mg/kg, 14 days, IP) increased weight gain, food intake, blood pressure, TG, LDL, blood sugar, leptin, and MDA in rat liver tissue and reduced GSH, AMPK, and P-AMPK in liver tissue in comparison with the control group. But, administration of alpha-mangostin decreased weight gain, food intake, systolic blood pressure, TG, LDL, blood sugar, leptin, MDA, and increased GSH, AMPK, and P-AMPK in liver tissue compared with the olanzapine group.

The results of a study carried out on 80 patients with schizophrenia disclosed that 66.6% of patients receiving olanzapine gained 1 to 5 kg over 4 weeks. The results of this study showed that weight gain was not related to drug dose or BMI. Interestingly, weight gain was significantly related to age and gender, and women over 40 years were more prone to weight gain with olanzapine compared with women under 40 years and men of any age (50). In another study on 25 healthy individuals, the effect of olanzapine was evaluated for one week. On the first night, they were given 5 mg/kg of olanzapine, and on subsequent nights, they were given 10 mg/kg. One week of olanzapine administration significantly increased weight gain and food intake (51). It has also been observed that chronic administration of olanzapine (100 mg/kg) in male and female rats has been associated with increased fat mass and weight gain in female rats (52). Moreover, it was illustrated that olanzapine is more likely to cause weight gain and metabolic disorders in female rats (24).

A study reported that oral administration of alpha-mangostin (50 mg/kg) for 6 weeks reduced body weight and fat mass in mice with a high-fat diet. These results suggest that alpha-mangostin may regulate lipid metabolism. Also, SirT1, AMPK, and PPAR pathways are thought to be involved in alpha-mangostin regulating hepatic steatosis and obesity in high-fat diet mice (41). In this regard, the findings of another study disclosed that alpha-mangostin inhibited cytoplasmic fat accumulation and lipid differentiation. 3T3-L1 fat cells treated with 50 µM alpha-mangostin dose-dependently reduced intracellular fat accumulation by 44.4% compared with cells treated with MDI (0.5 mM 3-isobutyl-1-methylxanthine (IBMX), 0.25 μM dexamethasone, and 1 μg/ml insulin) (49). In the present study, olanzapine (5 mg/kg) injection caused significant weight gain (from day 10), mean total food intake, and average food intake (from day 3) compared with the control group. Administration of alpha-mangostin along with olanzapine caused a significant weight loss, and reduced mean total food intake and average food intake compared with the olanzapine group. 

Leptin is produced and secreted by adipose cells and enterocytes and plays an important role in homeostasis, control of appetite, and energy regulation (53). Initially, most research on leptin focused on its role in regulating energy homeostasis and obesity at the central nervous system level. Due to association of leptin with changes in body weight and glucose metabolism, it is considered an indicator during treatment with atypical antipsychotic drugs (54). In a study on 13 schizophrenic patients treated with olanzapine for 4 weeks, serum leptin levels began to increase from week 4 (55). In an animal study on 24 female Wistar rats over 12 days, administration of olanzapine (5 mg/kg) increased leptin compared with controls (24). Since leptin decreases body weight and food intake, the synchronicity of raised leptin levels with obesity is generally interpreted as an indication of leptin resistance. Obesity stimulates several cellular processes that reduce leptin signaling which is known as cellular leptin resistance and increase the amount of weight gain prompted by environmental factors and genetics (56). Our data also revealed that the amount of leptin in the olanzapine group was significantly increased compared with the control group. Besides, in the present study, which examined the effect of alpha-mangostin on leptin level in an *in vivo* method for the first time, it was observed that intraperitoneal injection of alpha-mangostin (20, 40 mg/kg) along with olanzapine reduced leptin levels in rats. 

A study on mice showed that receiving olanzapine (3 mg/kg/day, 4 weeks) disrupted hepatic lipid metabolism, aortic cholesterol homeostasis, blood lipids, and increased the mean arterial pressure. Also, accumulation of lipids in the liver, especially cholesterol, fatty acids, and glycerol, and a decrease in HDL were observed in mice treated with olanzapine (13). Another study on 30 patients with schizophrenia showed that olanzapine increased weight, blood glucose, cholesterol, and visceral fat (57). In another study of 25 patients over 12 weeks, olanzapine increased weight and TG levels (58).

In a study on diabetic rats, oral administration of alpha-mangostin (25, 50, 100 mg/kg) for 28 days decreased blood glucose, TG, LDL, and increased plasma insulin and HDL levels (59). Another study on high-fat rats showed that oral administration of alpha-mangostin (50 mg/kg) for 6 weeks reduced accumulation of retroperitoneal fat, cholesterol, glucose, LDL, and serum TG (41). In the current research, olanzapine administration significantly increased TG, LDL, FBS, and systolic blood pressure in rats compared with the control group. Also, administration of alpha-mangostin significantly reduced blood TG and LDL levels. Also, all three doses of alpha-mangostin reduced blood cholesterol levels, but this reduction was not significant which might be due to the short duration of the study. In addition, administration of alpha-mangostin 40 mg/kg with olanzapine was able to significantly reduce FBS and systolic blood pressure.

Oxidative stress is a procedure that shows the negative effects of disturbing the balance between pro-oxidative and antioxidant factors (60). MDA is hypothesized to arise from lipid peroxidation and is an indicator of oxidative stress in tissues and cells (61). GSH is an endogenous peptide that can be synthesized in the liver and it is an important antioxidant that protects cells against oxidative damage by reacting with free radicals and peroxidase (47).

Obesity can cause oxidative stress due to an imbalance of pro-oxidants and antioxidants in the body (62). In this regard, a study showed that there is a direct relationship between leptin levels and MDA and an inverse relationship between leptin levels and GSH (63). It was also reported that administration of antipsychotic drugs to patients with schizophrenia increased serum levels of oxidative stress markers, including MDA (64). A study showed that olanzapine causes hepatotoxicity in isolated hepatocytes by increasing ROS and decreasing GSH levels (65).

A document on hepatic stellate cells showed that alpha-mangostin increased GSH levels (66). It has also been reported that oral administration of alpha-mangostin (100, 200 mg/kg, a week) to rats with acetaminophen-induced hepatotoxicity decreased MDA and increased GSH in liver tissue (67). In the current study, it was observed that olanzapine administration significantly increased MDA and decreased GSH in liver tissue and these changes were reversed by alpha-mangostin administration.

AMPK is an important enzyme in energy metabolism regulation. Increased fatty acid oxidation, glucose absorption, and glycolysis, inhibition of fatty acid synthesis, gluconeogenesis, and stimulation of mitochondrial biogenesis are all pleiotropic effects of its activation in several tissues. Recently, AMPK has also been recognized as an appetite regulator, helping to control energy metabolism at both the cell surface and the body as a whole. AMPK also appears to regulate hypothalamic glucose and nutrient levels (68). Also, in a study on female rats, it was found that oral administration of olanzapine (1 mg/kg three times a day for 36 days) significantly reduced P-AMPK protein in the hypothalamus (69).

Also, administration of alpha-mangostin (50 mg/kg) to rats on a high-fat diet has been shown to activate hepatic AMPK expression and cause weight loss (41). The findings of the current work also disclosed that administration of olanzapine reduced the levels of AMPK and P-AMPK, but co-administration of alpha-mangostin and olanzapine prevented this reduction.

## Conclusion

The results of the present study show that the administration of olanzapine increases weight gain, food intake, blood pressure, and elevates leptin, FBS, TG, and LDL in blood serum. Also, olanzapine causes oxidative stress by decreasing GSH and increasing MDA and induces metabolic disorders by inhibiting AMPK phosphorylation. However, the concomitant administration of olanzapine and alpha-mangostin causes weight loss, reduces food intake, systolic blood pressure, and decreases leptin, TG, LDL, and FBS levels. It also ameliorates oxidative stress by increasing GSH and decreasing MDA and alleviates olanzapine-induced metabolic disorders by increasing AMPK phosphorylation.

## Authors’ Contributions

HH and BMR Supervised the research and designed the experiments; AA Performed the experiments; FO and MGR Supervised the implementation of the experiments; all authors reviewed the results and approved the final version of the manuscript.

## Conflicts of Interest

 The authors declare that they have no conflicts of interest.
